# Case report of severe hypocalcemia with atypical symptoms after zoledronic acid in palliative care: a deprescribing pitfall

**DOI:** 10.1186/s12904-026-02070-8

**Published:** 2026-03-21

**Authors:** Till Arnold, J. Berner-Sharma, D. Rudolph, C. Bausewein, C. Rémi

**Affiliations:** 1 Department of Palliative Medicine, LMU University Hospital, Munich, Germany; 2 Klinik und Poliklinik für Palliativmedizin, Marchioninistr. 15, Munich, 81377 Germany

**Keywords:** Hypocalcemia, Nausea, Bronchospasm, Palliative, Palliative care, Deprescribing

## Abstract

**Background:**

Symptomatic hypocalcemia is an uncommon metabolic disturbance in palliative care and may therefore be overlooked. While deprescribing is a key principle in this setting, routine discontinuation of calcium and vitamin D supplementation may be harmful in selected high-risk patients, particularly after recent antiresorptive therapy.

**Case presentation:**

A 74-year-old woman with hormone receptor-positive metastatic breast cancer, extensive bone metastases and chronic kidney disease, was admitted to a German palliative care unit. Four weeks prior, she had received zoledronic acid, and calcium and vitamin D supplementation were discontinued on admission. She subsequently developed persistent nausea, vomiting and bronchospasm without classical neuromuscular signs, which were initially attributed to other causes. At a corrected calcium nadir of 0.95 mmol/L intravenous calcium was started and symptoms resolved. Oral intake resumed and supplementation was restarted. She was discharged to hospice care and died nine months later.

**Conclusion:**

After recent antiresorptive therapy, especially in patients with additional risk factors such as renal impairment, hypocalcemia may present with atypical symptoms including nausea or bronchospasm. This case highlights a potential pitfall of deprescribing in palliative care and underscores the need for individualized decisions regarding calcium and vitamin D supplementation to prevent delayed diagnosis and avoidable symptom burden.

## Background

Symptomatic hypocalcemia is an uncommon metabolic disturbance in palliative care and may therefore be overlooked. While medication deprescribing is an important principle in a palliative care setting [1], discontinuation of long-term supplements such as calcium and vitamin D may be harmful in selected high-risk situations. We report a case of severe hypocalcemia following recent zoledronic acid administration and discontinuation of calcium and vitamin D supplementation, presenting with atypical symptoms including bronchospasm and nausea in the absence of classical neuromuscular signs. This case highlights an underrecognized presentation of hypocalcemia and its implications for deprescribing decisions in palliative care.

## Case presentation

A 74-year-old woman with hormone receptor-positive metastatic breast cancer with extensive bone metastases and prior skeletal complications including spinal involvement and pathologic fractures, who had received palliative radiotherapy, was admitted to a German inpatient palliative care unit in a tertiary hospital for symptom control and care planning. The patient had declined systemic cancer therapy multiple times in the past. One month prior to admission, she consented to letrozole and zoledronic acid therapy to reduce bone-related events. Her extended medical history included atrial fibrillation, hypertension, hypothyroidism, and unilateral renal atrophy.

On admission, symptoms included pain, dry mouth, mild dyspnea, generalized weakness, and poor mobility reflected by an Australian-modified Karnofsky Performance Status (AKPS) of 30%. Laboratory results showed CKD, mild hypocalcemia, pancytopenia and mildly elevated CRP (Table 1).

**Table 1:** Laboratory values on admission and during the trajectory

Laboratory value	Patient values on admission	Day 7	Day 9	Day 10	Day 11	Day 13	Day 15	Reference range
Creatinine	1.1	1.3	1.5	1.5	1.1	0.9	0.8	0.5–1.0 mg/dL
GFR	49	40	34	34	49	63	73	≥ 60 mL/min/1.73 m²
Calcium	1.89	1.17	1.00	1.15	1.29	1.60	1.62	2.05–2.65 mmol/L
Albumin	3.8	3.8	4.2		3.7	3.7	3.8	3.5–5.2 g/dL
Albumin-corrected Calcium	1.94	1.22	0.95		1.37	1.68	1.67	2.05–2.65 mmol/L
Sodium	137	133	136	134	139	138	140	135–145 mmol/L
Potassium	3.9	3.0	3.3	4.1	3.4	3.7	4.2	3.5–5.1 mmol/L
CRP	0.7	1.7	1.0	0.7		0.6		≤ 0.5 mg/dL
Magnesium			1.06	1.11	1.19	1.24		0.66–1.07 mmol/L
PTH						162		15–65 pg/mL
25-OH-Vitamin D			38.2					20–100 ng/mL
Inorganic phosphate			8.2			4.9		2.5–4.8 mg/dL
Alkaline phosphatase			316					35–105 U/L
LDH		392	517	517	457			≤ 249 U/L
Leukocytes	3.04	3.79	5.74	5.86			5.15	4.00–10.40 G/L
Hemoglobin	8.6	8.7	9.5	8.1			9.3	11.5–15.4 g/dL
Thrombocytes	143	186	241	229			217	176–391 G/L

Pre-admission oral medications included calcium 1,000 mg and vitamin D 1,000 IU once daily, as well as a proton pump inhibitor, loop and thiazide-like diuretics, and other chronic medications. In line with the patient’s wishes, calcium/vitamin D and other non-essential drugs were discontinued. Diuretics were continued, and pain control was optimized with rotation from an oral opioid combination to a transdermal opioid, leading to overall clinical stabilization.

A symptomatic catheter-associated urinary tract infection 4 days after admission was treated empirically with ciprofloxacin and the catheter was replaced. The following day, the patient started to develop nausea and vomiting. Despite switching antibiotics to cotrimoxazole and symptomatic treatment with metoclopramide, nausea and vomiting persisted. Positional vertigo was considered, and hydromorphone was administered prior to positional testing due to mobilization pain. Shortly thereafter, the patient developed dyspnea with inspiratory stridor, somnolence, and worsening nausea, with oxygen saturation falling below 80% on room air. No allergic, infectious or opioid intoxication signs were found. ECG revealed QTc prolongation of 480 ms. Point-of-care ultrasound showed no pleural effusion or pneumothorax and no signs suggestive of right heart strain.

Laboratory results revealed worsening renal function and a marked drop in albumin-corrected calcium (Table 1, day 7). Our initial clinical suspicion focused on early aspiration pneumonia, and symptomatic treatment was initiated. To treat bronchospasm, salbutamol and ipratropium bromide were prescribed by inhalation, along with intravenous dexamethasone 4 mg once daily for three days. For nausea relief, metoclopramide 5 mg was administered three times daily.

However, over the next two days, symptoms worsened, oral intake became impossible, and signs of terminal decline emerged. Further laboratory testing on day 9 ruled out infection but revealed severe albumin-corrected hypocalcemia (0.95 mmol/L), hyperphosphatemia, mildly elevated magnesium, normal 25-OH-vitamin D, hyponatremia and worsening of kidney function (Table 1). Despite the absence of tetany or paresthesias, a therapeutic trial with intravenous calcium gluconate was initiated. First, potassium replacement was initiated with 40 mEq added to 1,000 mL of crystalloid solution. Then two intravenous boluses each of 10 mL calcium gluconate 10% in 50 mL NaCl 0.9% over 1 h were administered, followed by a continuous infusion of 100 mL calcium gluconate 10% in 1000 mL NaCl 0.9% over 24 h. Shortly after initiating the infusion, the patient developed palpitations and new-onset atrial fibrillation, which was managed successfully with 5 mg intravenous metoprolol. As corrected calcium rose, dyspnea and nausea improved and oral intake resumed; oral calcium/vitamin D was restarted. Parathyroid hormone (PTH) was elevated to 162 pg/mL, suggesting secondary hyperparathyroidism due to functional calcium deficiency. The patient was discharged in stable condition to hospice care. The patient died nine months later.

## Discussion and conclusion

### Pathogenesis and epidemiology of hypocalcemia

Serum calcium homeostasis is regulated primarily by parathyroid hormone (PTH), vitamin D, calcium ions themselves, and phosphate. Hypocalcemia most commonly results from disturbances in PTH secretion or vitamin D metabolism, but may also occur due to renal failure, hyperphosphatemia, osteoblastic bone metastases, or severe systemic illness [2–4]. Approximately 40–45% of circulating calcium is protein-bound, predominantly to albumin, while only the ionized fraction is biologically active [5]. In patients with hypoalbuminemia, albumin-corrected or ionized calcium should therefore be assessed to avoid pseudohypocalcemia [6, 7].

We were unable to identify any literature on the incidence or prevalence of clinically relevant hypocalcemia in specialist palliative care settings. Its pooled prevalence among all inpatients is about 18%, with a higher number of 85% in intensive care patients [2, 5]. In cancer patients, its prevalence is around 67% but seems to have no influence on survival times [8]. Even though hypocalcemia is frequent in patients with advanced diseases and may have severe consequences, it is not reported as often as hypercalcemia, which is found in up to 41.3% of patients with advanced malignancy [9]. This might be due to an oligosymptomatic presentation of hypocalcemia in many cases, while hypercalcemia is often symptomatic [9].

In cancer patients receiving bone-modifying agents (BMA) such as bisphosphonates or denosumab, hypocalcemia is a recognized adverse effect, with reported incidences ranging from 6% to 35% [10–12], and severe cases occurring in up to 9% [11]. Table 2 summarizes identified risk factors for the development of hypocalcemia in patients receiving BMA.

Other medications may also contribute to hypocalcemia, including calcium-binding agents such as citrate, bisphosphonates, cinacalcet, foscarnet, fluoride intoxication, chemotherapeutic agents such as cisplatin, or immune checkpoint inhibitors such as nivolumab [4, 13–15].

Renal impairment is frequent in palliative patients and has been found to increase the risk of hypocalcemia in combination with BMA [10, 14]. This is caused by multiple mechanisms that include a reduced absorption of calcium from the gastrointestinal tract because of a reduced production of 1,25-(OH)_2_ vitamin D, lower levels of 25(OH) vitamin D and binding of calcium due to hyperphosphatemia [16]. This is normally compensated by a secondary hyperparathyroidism which becomes disturbed by BMA [4, 16], resulting in a higher risk of hypocalcemia.

**Table 2:** Risk factors of hypocalcemia related to bone-modifying agents

Category	Risk factor	Medication implicated	Reference
Patient/disease related	Low pretreatment vitamin D	Denosumab, zoledronic acid	[11]
	Low pretreatment calcium	Denosumab	[12, 14]
	Renal impairment	Denosumab, zoledronic acid	[10, 14]
	Hematologic malignancy and/or bone metastases	Denosumab, zoledronic acid	[11]
	Elevated alkaline phosphatase	Denosumab	[12, 17]
	Age ≥ 65, male sex	Denosumab	[12, 14]
	Low haemoglobin	Denosumab	[17]
Therapy related	Concomitant use of dexamethasone or vonoprazan	Denosumab	[17]
	Concomitant use of loop diuretics, aminoglycosides, calcitonin	Zoledronic acid	[18]
	Co-administration of cytotoxic agents	Denosumab	[12]
Electrolyte/metabolic	Hypomagnesaemia	Denosumab, zoledronic acid	[10, 14]
	Hypophosphatemia	Denosumab	[14]

### Symptoms and diagnosis of hypocalcemia

Hypocalcemia may be asymptomatic or present with a wide range of clinical manifestations, depending on severity and acuity. Acute hypocalcemia typically causes neuromuscular irritability and cardiovascular symptoms. A number of clinical signs such as Trousseau or Chvostek indicate this excitability [4, 19]. Less common symptoms such as bronchospasm [20] and psychiatric manifestations [21] have also been reported. Table 3 shows clinical findings in hypocalcemia.

Gastrointestinal symptoms are rarely emphasized in the literature. Nausea has only sporadically been described as a manifestation of hypocalcemia [15]; however, in our case it was the leading symptom and resolved promptly after calcium replacement, suggesting a causal relationship. We therefore believe it should be included among atypical symptoms of hypocalcemia.

Diagnosis relies on laboratory assessment of albumin-corrected or ionized calcium, with additional evaluation of PTH, vitamin D, phosphate, magnesium, and renal function to clarify the underlying mechanism [22, 23].

**Table 3:** Symptoms of acute and chronic hypocalcemia

Acute hypocalcemia		Reference
Neuromuscular irritability	Paresthesias (perioral and extremities) Muscle twitching, cramps and weakness Carpopedal spasm Tetany Seizures (focal, petit mal, grand mal) Laryngospasm Bronchospasm	[4, 5, 19] [4, 5] [4, 5] [4] [4] [4] [4, 20]
Cardiac	Prolongation of QTc interval Hypotension Heart failure Arrhythmia	[4, 19, 24] [2] [4, 24] [25]
Neuropsychiatric	Confusion Anxiety Irritability and agitation Psychosis	[4] [21] [4, 26] [26]
Ocular	Papilledema	[4]
Gastrointestinal	Nausea Vomiting Abdominal pain	[15] [15] [4]

Chronic hypocalcemia		Reference
Neurologic	Basal ganglia calcifications / Extrapyramidal disorders (Fahr disease) Cognitive decline / dementia	[4, 19] [19, 27]
Ocular	Subcapsular cataracts Papilledema Optic neuritis	[5, 28] [4] [29]
Ectodermal / dental	Alopecia Xeroderma Brittle nails, dry skin Enamel hypoplasia / altered tooth morphology	[4] [4] [4] [4]

### Management of hypocalcemia

Management of hypocalcemia depends on symptom severity, acuity, and the underlying cause. Acute symptomatic hypocalcemia typically requires intravenous calcium administration under inpatient monitoring [30], whereas chronic or asymptomatic cases may be managed with oral supplementation [31]. Intravenous therapy is typically administered as repeated boluses followed by continuous infusion if needed, aiming to restore calcium levels to the low-normal range [4]. Oral calcium and vitamin D supplementation should be initiated as soon as clinically feasible.

Potential complications of intravenous calcium include arrhythmias, especially after rapid administration, local vein irritation (in solutions with more than 200 mg/100 mL of elemental calcium), and tissue calcification may result from extravasation [4]. Compensating for any hypomagnesemia to overcome possible PTH resistance needs to be considered [4].

### Synopsis and relevance in palliative care

Deprescribing is the structured, supervised process of withdrawing medications that are no longer beneficial or may be harmful, with the goal of improving outcomes aligned with patient priorities. In palliative care, it is essential to reduce pill burden, adverse effects, and treatment complexity, particularly when prognosis is limited or symptoms and goals of care change over time [1]. This case highlights an important pitfall of deprescribing in palliative care, where discontinuation of long-term therapies may have unintended clinical consequences. While deprescribing is a key principle in palliative care, routine cessation of supplements such as calcium and vitamin D may be harmful in selected high-risk situations (Table 2). In our patient, recent zoledronic acid therapy, pre-existing renal impairment, and low baseline calcium levels constituted a constellation in which continued supplementation was clinically relevant.

The decision to deprescribe calcium/vitamin D was made in accordance with the patient’s wishes and common palliative practice, yet the potential delayed effects of antiresorptive therapy were underestimated. This illustrates how the benefits and risks of deprescribing may shift after specific oncological treatments, even when these treatments were administered weeks earlier [32].

Hypocalcemia may be particularly difficult to recognize in palliative care, where symptoms are often multifactorial and laboratory monitoring may be limited. In this context, atypical manifestations such as nausea or bronchospasm can easily be misattributed to disease progression, infection, medication side effects, or terminal decline. Failure to recognize hypocalcemia may lead to unnecessary interventions and delayed symptom relief, and inappropriate therapeutic decisions that may even exacerbate the underlying problem, such as the administration of dexamethasone.

This report has several limitations inherent to a single-case design and does not allow causal inference. Ionized calcium was not measured, and symptom attribution remains challenging in a palliative care context with multiple potential confounders (e.g., infection, opioid rotation, and concomitant medications). Moreover, oral intake and adherence to supplementation prior to admission could not be verified. Nevertheless, the temporal relationship between discontinuation of calcium/vitamin D after recent BMA exposure, biochemical findings, and rapid symptom improvement following calcium repletion supports hypocalcemia as a clinically relevant contributor to symptom burden.

Our case underscores the need for individualized deprescribing decisions rather than blanket discontinuation of supplements. In patients with recent exposure to bisphosphonates or denosumab, especially those with additional risk factors, calcium and vitamin D supplementation should generally be continued if oral intake is preserved and prognosis allows. In oncologic patients receiving BMA, it is even recommended to check calcium levels before each dose and two weeks after the first dose for patients with risk factors [18, 32]. Awareness of this potential complication may help prevent avoidable suffering and misinterpretation of symptoms in palliative care settings.

### Home and hospice care perspective

In Germany, specialist palliative home care (spezialisierte ambulante Palliativversorgung, SAPV) has become an important pillar of palliative care for patients wishing to stay at home [33]. Other patients stay in hospices at the end of their lives. In these settings, hypocalcemia may be easily overlooked, particularly if symptoms are atypical such as nausea or bronchospasm. If a patient on antiresorptive therapy develops new or unexplained symptoms, serum calcium should be checked if feasible. Oral calcium and vitamin D supplementation can be safely managed at home. If intravenous calcium is necessary and indicated, referral to hospital or a specialized palliative care unit remains the safest approach. Only in carefully selected situations, with a confirmed diagnosis and explicit patient consent, could intravenous therapy be considered at home when hospital admission is not an option. Our case underlines the importance of such vigilance: early recognition and appropriate management of hypocalcemia, even when presenting with atypical symptoms such as nausea, may prevent misdiagnosis and unnecessary interventions in both inpatient and palliative home care.

## Data Availability

Data sharing is not applicable to this article as no datasets were generated or analysed during the current study.

## Abbreviations

BMA: Bone modifying agent(s)
CKD: Chronic kidney disease
CRP: C-reactive protein
ECG: Electrocardiogram
GFR: Glomerular filtration rate
IU: International units
IV: Intravenous
LDH: Lactate dehydrogenase
PTH: Parathyroid hormone
QTc: Corrected QT interval
SAPV: specialist palliative home care (*Spezialisierte ambulante Palliativversorgung*)
